# Nanostructured SmFeO_3_ Gas Sensors: Investigation of the Gas Sensing Performance Reproducibility for Colorectal Cancer Screening

**DOI:** 10.3390/s20205910

**Published:** 2020-10-19

**Authors:** Andrea Gaiardo, Giulia Zonta, Sandro Gherardi, Cesare Malagù, Barbara Fabbri, Matteo Valt, Lia Vanzetti, Nicolò Landini, Davide Casotti, Giuseppe Cruciani, Michele Della Ciana, Vincenzo Guidi

**Affiliations:** 1 MNF—Micro Nano Facility, Bruno Kessler Foundation, Via Sommarive 18, 38123 Trento, Italy; vanzetti@fbk.eu; 2 SCENT S.r.l., Via Quadrifoglio 11, 44124 Ferrara, Italy; giulia.zonta@unife.it (G.Z.); gherardi@fe.infn.it (S.G.); malagu@fe.infn.it (C.M.); nicolo.landini@unife.it (N.L.); 3 Department of Physics and Earth Sciences, University of Ferrara, Via Saragat 1, 44122 Ferrara, Italy; fbbbbr@unife.it (B.F.); matteo.valt@unife.it (M.V.); giuseppe.cruciani@unife.it (G.C.); dellaciana@bo.imm.cnr.it (M.D.C.); 4 INFN Section of Ferrara, Via Saragat 1/c, 44122 Ferrara, Italy; cstdvd@unife.it; 5 CNR-NANO, via G. Campi 213/a, 41125 Modena, Italy; 6 Unit of Bologna, Institute for Microelectronics and Microsystems, National Research Council, via Gobetti 101, 40129 Bologna, Italy

**Keywords:** nanostructured SmFeO_3_ perovskite, chemoresistive gas sensors, gas sensor reproducibility, CRC screening, medical diagnostic tool

## Abstract

Among the various chemoresistive gas sensing properties studied so far, the sensing response reproducibility, i.e., the capability to reproduce a device with the same sensing performance, has been poorly investigated. However, the reproducibility of the gas sensing performance is of fundamental importance for the employment of these devices in on-field applications, and to demonstrate the reliability of the process development. This sensor property became crucial for the preparation of medical diagnostic tools, in which the use of specific chemoresistive gas sensors along with a dedicated algorithm can be used for screening diseases. In this work, the reproducibility of SmFeO_3_ perovskite-based gas sensors has been investigated. A set of four SmFeO_3_ devices, obtained from the same screen-printing deposition, have been tested in laboratory with both controlled concentrations of CO and biological fecal samples. The fecal samples tested were employed in the clinical validation protocol of a prototype for non-invasive colorectal cancer prescreening. Sensors showed a high reproducibility degree, with an error lower than 2% of the response value for the test with CO and lower than 6% for fecal samples. Finally, the reproducibility of the SmFeO_3_ sensor response and recovery times for fecal samples was also evaluated.

## 1. Introduction

Nowadays, the market for solid-state gas sensors is constantly growing, as they are used in a wide variety of applications, including indoor and outdoor air quality monitoring, medical screening and precision farming [1,2,3,4]. Solid-state gas sensors are divided in four different broad categories, i.e., chemoresistive, optical, electrochemical gas sensors and quartz microbalance [5]. Among them, chemoresistive gas sensors based on metal oxide (MOX) semiconductors are the most investigated, because of their great versatility [6]. They are devices characterized by high sensitivity towards gas concentration changes (up to tens of ppb for some gases), long-term repeatability, low cost and the possibility of mass production through microfabrication processes [6,7]. Nevertheless, the widespread use of the MOX gas sensors is still limited by some of their shortcomings, such as poor selectivity and the baseline drift [8,9]. In the last few years, the research followed two pathways to overcome these limitations. On the one hand, artificial intelligence and new methods of data analysis, e.g., machine learning, were used to improve the MOX sensing performances [10]. In particular, specifically calibrated arrays of MOX gas sensors equipped with a dedicated algorithm have proven to be suitable for different applications [11]. On the other hand, great effort has been devoted to the study and development of advanced nanostructured sensing materials, by considering both MOX and other types of semiconductors [7,12,13,14,15]. The most used techniques were based on the addition of catalysts to the MOX nanostructure surface, on the synthesis of different MOX morphologies and on the preparation of MOX solid solutions [6,7,16]. Specifically, the MOX solid solutions showed very good sensing performances due to the high tunability of their physical and chemical properties, which can be controlled during the synthesis process by modifying synthesis parameters and the types and amounts of metals [17]. Among the various solid solutions, SmFeO_3_ perovskite is one of the most investigated [18,19]. It is a p-type semiconductor, which can be synthesized with simple and low-cost methods, e.g., sol–gel synthesis or co-precipitation in water solution [20]. Several works have highlighted its excellent sensing properties, especially for the detection of oxidizing gases such as NO_2_ and O_3_ [21,22,23]. Furthermore, SmFeO_3_ has shown interesting sensing behaviors also in specific applications. In particular, it has demonstrated good sensing performances on the detection of volatile biomarkers exhaled by colorectal cancer (CRC) during the SCENT prototype validation protocol [4]. Therefore, it is a strong candidate to be used in the SCENT sensor array for the CRC screening [3].

To develop sensing platforms useful for commercial purposes, it is essential to obtain sensors with high degrees of reproducibility, to ensure the same responses to the same analytes in different devices of the same type. In order to enhance reproducibility, it is fundamental to work on the optimization of the fabrication method of the sensing layer. Subsequent thermal treatments, the homogeneity of nanostructures and the deposition of the paste are key aspects to obtain reproducible gas sensing devices [24]. Reproducibility is fundamental, particularly when sensors are employed for medical devices [3,25] because in these instruments response signals have to be processed by specific software based on machine learning techniques such as principal component analysis (PCA) or the support vector machine (SVM) [26,27], to reach a high discrimination capability for the target samples. These algorithms are strongly related to sensors’ structural characteristics and the sample datasets used for calibration measured with specific devices, composed by their own sensing cores [3]. In order to use the same software for different devices of the same type, what needs to be done is the estimation of a minimum error margin which does not undermine the discriminatory capacity.

In this work, the response reproducibility of a set of four thermo-activated sensors based on a SmFeO_3_ perovskite sensing layer was tested to verify their employability in medical diagnostic tools for the CRC screening. It was decided to focus our attention on the investigation of the sensing response reproducibility of nanostructured SmFeO_3_ because, among the various sensing materials tested during the medical trial, it showed the best sensing properties on the detection of CRC in fecal samples, together with the (Sn-Ti)O_2_ (ST20) sensing material [3,4,25]. The SmFeO_3_ nanopowder was synthesized through a simple and low-cost procedure, which enables the preparation of the most suitable SmFeO_3_ layer for the above-mentioned application. Reproducibility was tested by exposing the SmFeO_3_ sensors both to defined concentrations of CO (gas that we generally employ for devices calibration), in a dedicated gas test chamber, and to fecal samples, by placing the SmFeO_3_ sensors in a portable device better described in the following section. In the latter test, biologic sample exhalations were sent to sensors to state sensor reproducibility for CRC preventive screening. The response and recovery time reproducibility for the SmFeO_3_ sensors against fecal samples was also evaluated, since these parameters are considered in the SCENT prototype algorithm, and the sensing response value [3]. This algorithm, based on the analysis of sensor response parameters by means of support vector machine (SVM) [26], was designed starting from a single device calibration to be adapted to multiple devices of the same type. The results shown in this manuscript are fundamental to demonstrating the reproducibility of SmFeO_3_ sensors, one of the sensing materials employed in SCENT devices. Reproducibility is the essential characteristic for the production of devices based on a common specific algorithm. In fact, calculating and considering deviation in the reproducibility of these parameters, it is possible to develop a dedicated software for CRC screening that can compensate for slight differences between gas sensors due to lack of reliability in the preparation process of the chemoresistive gas sensors [3].

## 2. Materials and Methods

### 2.1. SmFeO_3_ Synthesis, Film Deposition and Characterization

The nanostructured SmFeO_3_ perovskite was prepared through the thermal decomposition of Sm(Fe(CN)_6_) × 4H_2_O heteronuclear complex. All the chemicals were from Sigma Aldrich. Sm(Fe(CN)_6_) × 4H_2_O was synthesized by mixing an equimolar amount of Sm(NO_3_)_3_ × 6H_2_O and K_3_Fe(CN)_6_ ([Sm^3+^] = [Fe^3+^] = 0.1M) in 200 mL of deionized water. The solution was kept under continuous stirring for 4 h. The resulting precipitate was filtered from the water and washed with deionized water and diethyl ether. The powder was then dried at 50 °C for 12 h. Finally, SmFeO_3_ oxide powder was obtained by the thermal decomposition of the complex at 700 °C for 2 h.

The X-ray diffraction (XRD) analysis was carried out by using a Bruker D8 Advance diffractometer (Bruker AXS GmbH, Karlsruhe, Germany) equipped with an X-ray tube operating at 40 kV and 40 mA, and with a Si(Li) solid-state detector (SOL-X) set to discriminate the Cu Kα1,2 radiation. The EVA version 10. 0. 1. 0 program by Bruker AXS GmbH, coupled with the Powder Diffraction File database (PDF) version 9.0.133, was used for phase identifications. The Rietveld method, implemented in the TOPAS version 4.1 program by Bruker AXS GmbH [28], was used to calculate the cell parameters and the average crystallite size. The line profile fitting was carried out with the fundamental parameters approach [29,30,31,32]. The determination of crystallite size by TOPAS was accomplished by the Double–Voigt approach [33,34].

The SmFeO_3_ nanopowder morphology was investigated by using a Zeiss Field Emission Gun-Scanning Electron Microscope (FEG-SEM) LEO 1530 (Zeiss, Oberkochen, Germany) at 5.00 kV equipped with a hot cathode field emission column emitter and an energy Dispersive X-ray spectrometer (EDX).

X-ray photoelectron spectroscopy (XPS) measurements were performed using a Kratos AXIS Ultra^DLD^ instrument (Kratos Analytical, Manchester, UK) equipped with a hemispherical analyzer and a monochromatic Al Kα (1486.6 eV) X-ray source, in spectroscopy mode. The SmFeO_3_ sample was analyzed with a take-off angle between the analyzer axis and the normal to the sample surface of 0°, corresponding to a sampling depth of approximately 10 nm. Initially, a survey (in the 1300, −5 eV energy range) was recorded to identify the elements present on the surface and subsequently Sm 3d_5/2_, Fe 2p, O 1s and C 1s core levels were acquired with higher energy resolution. Charge compensation was achieved using a charge neutralizer located at the bottom of the electrostatic input lens system. The spectra were aligned setting C 1s core level hydrocarbon peak at 285 eV. The quantification, reported as relative elemental percentage, was carried out using the core levels and the atomic sensitivity factors. All XPS data were analyzed using the software described in Speranza and Canteri [35].

The SmFeO_3_ nanopowder was mixed with organic vehicles in order to obtain a printable paste [20]. Afterwards, the gas sensing material was deposited over alumina substrates by means of screen-printing techniques [12,24]. The alumina substrates were equipped with interdigitated gold electrodes on the top-side and platinum heaters on the back-side of the substrates [24]. The thickness of the deposition was about 20–30 µm [13].

### 2.2. Experimental Setups

**Figure 1:**
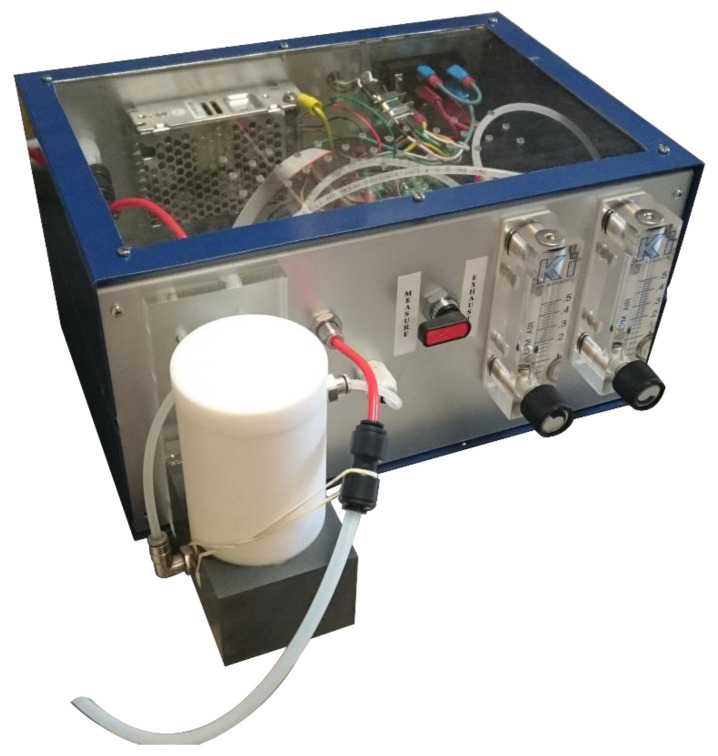
SCENT A2 external view.

In this work, two different experimental setups were employed. The first was a laboratory setup, composed of mass-flow controllers for gas mixing (MKS), power suppliers (Aim TTi), multimeter (K2000 (Keitheley)), Teflon tube lines, a data acquisition unit with specific software realized in LabVIEW and a hermetically sealed chamber of cylindrical shape (diameter: 139 mm, height: 41 mm). At the center of the chamber a gas diffuser was placed, while sensors were positioned circularly around it. Inside the chamber it was possible to place up to eight sensors, together with a humidity sensor and a temperature sensor. Furthermore, it is important to specify that the gas was diffused uniformly inside the chamber in order to solicit sensors at the same way. The second experimental setup was a portable prototype named SCENT A2 (Scent s.r.l., Ferrara, Italy). This was the second prototype of a patented device [36] employed in a three-year clinical validation protocol [3,4], ended in July 2019, for CRC preventive screening through the analysis of fecal exhalations [37]. Fecal exhalation may contain specific tumor volatile biomarkers, produced by cell membrane peroxidation or metabolic alterations. The prototype scheme, already described in our previous works [34] is simple and compact (Figure 1), and composed of a microfluidic system, a specific electronic, a sensing core with four sensor allocations (diameter 7.8 mm, height 25 mm) and a sample box.

A Laboratory system was employed for sensor calibration, in order to state the reproducibility of measurements with a standard gas (CO); after that, SmFeO_3_ sensors were inserted into SCENT A2 device, to test them directly on biologic samples.

## 3. Results and Discussion

### 3.1. Sensors Characterization

**Figure 2:**
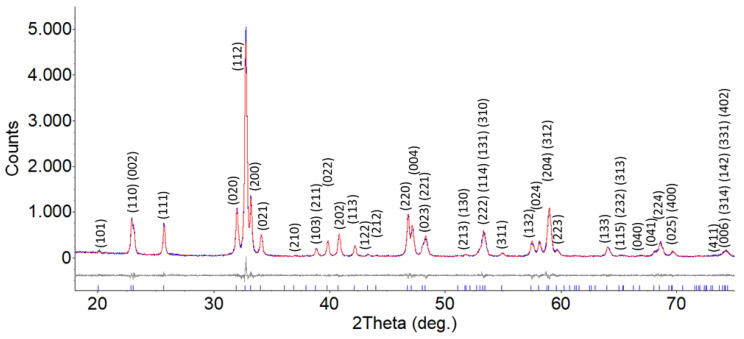
XRD analysis of the synthesized SmFeO_3_ powder. Planes’ indices are indicated in the graph alongside relative peaks. The spectrum highlights the presence of the Pbnm space group (pdf 01-074-1474).

The XRD pattern of the SmFeO_3_ nanopowder is shown in Figure 2. The only crystal phase identified in the sample is ascribable to SmFeO_3_, as space group Pbnm (PDF database: 01-074-1474). The phase composition analysis, as described in Section 2, highlighted the high purity of the SmFeO_3_ produced, and no other crystal phases due to contaminants were found in the sample. The crystallite size, calculated by the Rietveld method, was 38.34 ± 1.13 nm.

The lattice parameters of SmFeO_3_ samples are listed in Table 1.

**Figure 3:**
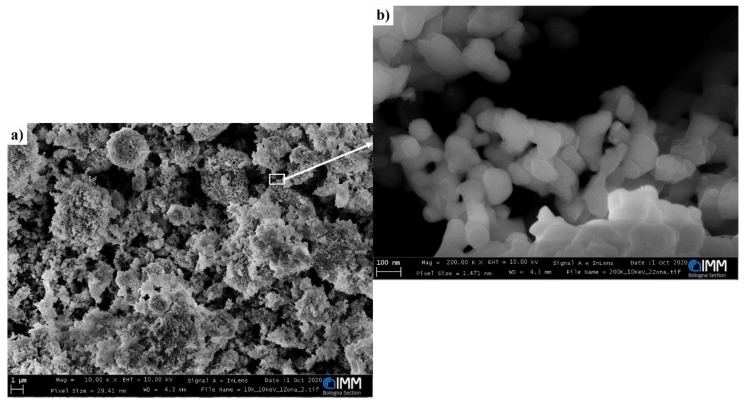
SEM images of the SmFeO_3_ nanoparticles with magnifications of (**a**) 10 kx and (**b**) 200 kx.

SEM images of the SmFeO_3_ nanopowder are shown in Figure 3. The material was deposited and pressed over a carbon tape placed over a silicon holder. As can be observed in Figure 3a, the deposition on carbon tape resulted in the formation of a porous film, with porosity similar to that obtained in screen-printed films of other nanostructured MOX [24]. The analysis highlighted that the morphology of SmFeO_3_ nanoparticles was spherical-like, with an average size of 40–50 nanometers (Figure 3b).

The chemical composition of the SmFeO_3_ nanopowder was investigated by using EDX analysis (Table 2). It can be noticed that the SmFeO_3_ sample showed a high chemical purity, apart from the presence of a low concentration of carbon, probably due to the use of carbon tape in the sample holder.

**Figure 4:**
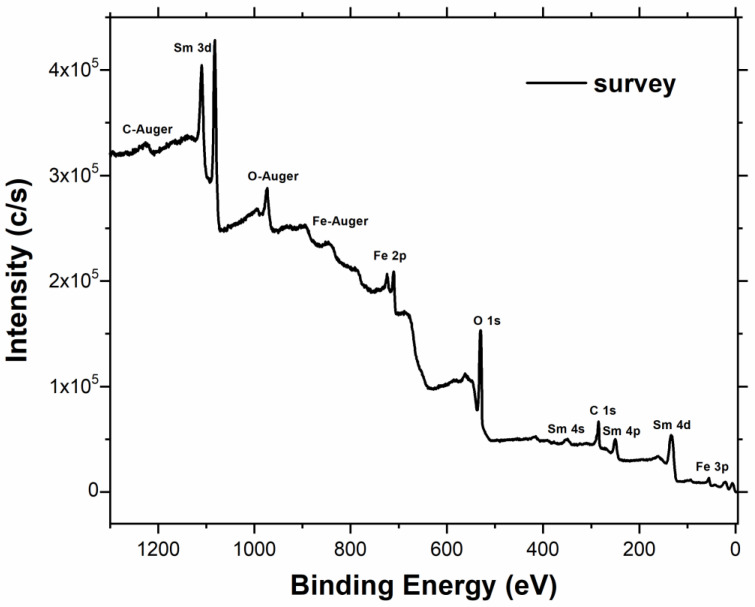
Survey of the SmFeO_3_ powder with the peak assignment.

The XPS survey of the SmFeO_3_ powder is shown in Figure 4, with the peak assignments. Besides samarium, iron, oxygen and carbon, there are no other elements on the surface of the sample, highlighting the purity of the SmFeO_3_ sample. The presence of carbon, previously observed also in the EDX characterization, was possibly due to the carbon tape on which the SmFeO_3_ nanopowder was deposited for the XPS analysis.

Table 3 shows the quantitative results for samarium, iron, oxygen and carbon, obtained from the core levels and using the atomic sensitivity factors.

The samarium content was more than two times that of iron. However, the XRD sample analysis (Figure 2) highlighted the presence of a SmFeO_3_ crystal phase related to a Sm/Fe ratio very close to 1, as also observed in the EDX characterization (Table 2). It probably means that Sm tends to segregate in first layers of the SmFeO_3_ nanoparticle surfaces, whereas in the nanoparticle bulks the solid solution lattice is composed of a stoichiometric SmFeO_3_. The oxygen quantification was affected by the presence of a very broad samarium Auger structure, peaked at around 680 eV.

**Figure 5:**
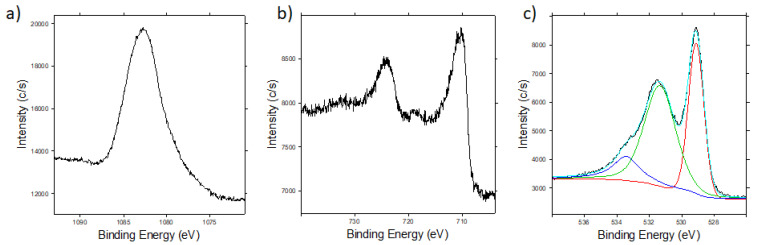
(**a**) Samarium Sm 3d_5/2_, (**b**) iron Fe 2p and (**c**) oxygen O 1s core levels of the SmFeO_3_ powder deposited on the carbon tape.

In Figure 5, Sm 3d_5/2_, Fe 2p and O 1s core levels are shown.

Fe 2p shows the typical line shape and binding energies of iron in Fe_2_O_3_. The peak of Sm 3d_5/2_ is located at the energy of samarium in Sm_2_O_3_, even though there is a small shoulder at lower binding energy that might be due to SmO or Sm [38].

Figure 5c shows the oxygen core level O 1s, with a fitting of the components. The peak at lower binding energy is probably due to O–Fe bonds, while the second peak is a mixture of O–Sm and O–C bonds; the latter probably belongs to the carbon tape. The shoulder at higher binding energy is possibly due, at least in part, to the broad Auger samarium structure above mentioned.

### 3.2. Sensor Calibration with CO

To test the reproducibility of SmFeO_3_ sensors, a set of four SmFeO_3_ sensors (here named S1, S2, S3, S4), obtained from the same screen-printing deposition, was introduced into the measurement chambers of laboratory setup. The gas employed for this first calibration was CO, and the measurements were carried out with a constant flux of 0.5 L/min. Due to the fact that we are dealing with a p-type material, responses (R) are calculated as the reciprocals of *G_gas_/G*, where *G_gas_* is the conductance with VOCs-contaminated air and *G* the conductance in environmental air.

**Figure 6:**
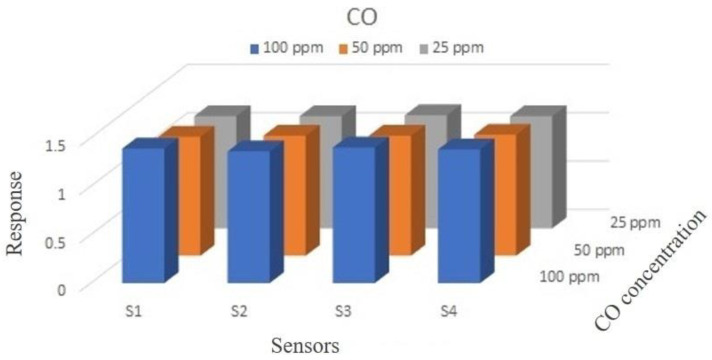
Histogram of the responses of the four SmFeO_3_ sensors, S1, S2, S3 and S4 (T = 350 °C) to 25, 50 and 100 ppm of CO.

All the measurements were performed by setting the SmFeO_3_ sensors at their best working temperature, i.e., 350 °C [4,23]. The CO concentrations analyzed were 100, 50 and 25 ppm respectively, chosen according to the threshold limit value [39]. The gas measurement was carried out in dry air (RH% < 1%, T = 22.3 °C). In Table 4, responses and their differences from mean response value (<R>) are reported for the three gas concentrations selected. To state reproducibility, a comparison between |R − <R>| of each sensor and R has been performed. What emerged is that the value |R − <R>| is lower than 2% of R for 100 ppm of CO and lower than 1% for 50 and 25 ppm of CO. Figure 6 is a histogram of responses to CO for the set of four sensors employed.

**Figure 7:**
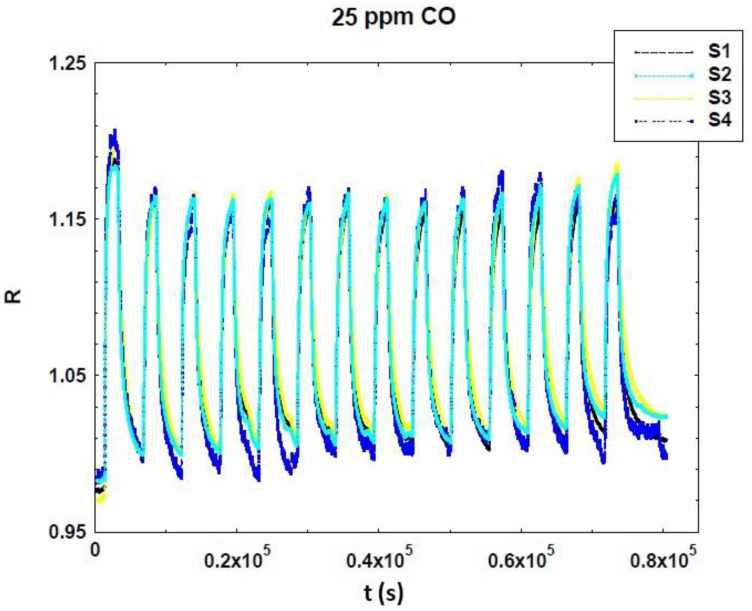
Normalized dynamic responses of SmFeO_3_ sensors vs. 25 ppm of CO, at a working temperature of 350°C.

In Figure 7 the dynamical response curve vs. time is reported for CO at 25 ppm. The measurement was repeated fourteen times in order to verify the repeatability of sensors’ responses. After that, the data were taken considering the second peak—the first one probably being altered by the line not being completely clean (e.g., presence of humidity and residuals of previous measurements).

### 3.3. Tests with Feces

After CO calibration in the laboratory setup, wherein sensors’ responses were reproducible below the 2% of R, as demonstrated in Section 3.2, the four sensors were inserted into SCENT A2 system, to state whether reproducibility is confirmed also with fecal samples. Fecal samples, a total of eight, were all of patients who tested positive on the fecal occult blood test (FOBT), before being tested with colonoscopy. These samples came from the clinical validation protocol of another device, SCENT A1 [3,4], for CRC screening through fecal exhalation analysis. Here the true health status of patients is not reported for privacy reasons and due to the fact that it is irrelevant for reproducibility tests. However, the different compositions of exhalations are evident from the response differences.

In Table 5 the same analysis on responses, already performed with CO, is reported. The measurements were carried out with 20% RH%, at a temperature range of 21 °C (accuracy of the Honeywell RH%/temperature sensor = ± 3.5%). The gas flux injected in the gas sensing chamber was 0.2 L/min for all the measurements.

The fecal samples, already tested the FOBT, were listed by assigning a progressive letter to them (from FOBT A to FOBT H). Average response values <R> are respectively the following: 1,28 (FOBT A); 2,11 (FOBT B); 1,51 (FOBT C); 1,58 (FOBT D), 2,29 (FOBT E); 1,52 (FOBT F); 1,73 (FOBT G); 1,30 (FOBT H).

**Figure 8:**
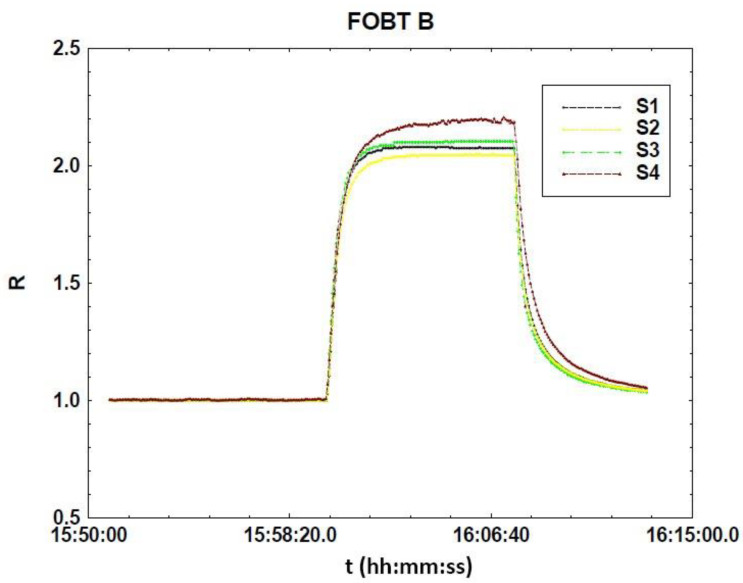
Normalized dynamic responses of SmFeO_3_ sensors vs. FOBT B exhalation, at a working temperature of 350 °C.

All |R − <R>| values stayed below 6% of R for all sensors, and below 3% for two fecal samples, demonstrating a high reproducibility degree. In Figure 8 the dynamic response curve of FOBT B is reported as an example to show the stability of both the baseline and the response plateau.

**Figure 9:**
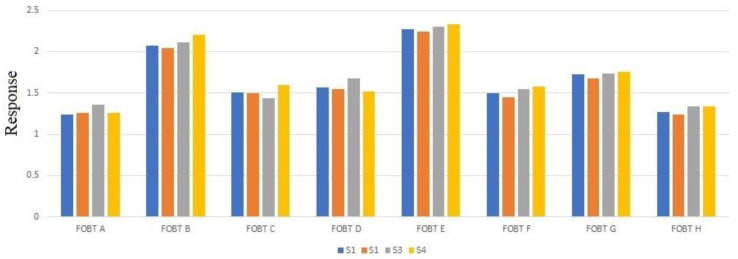
A histogram that compares the four sensor (S1–S4) responses for the eight FOBT-positive fecal samples analyzed.

The histogram reported in Figure 9 is a summary of all sensor responses analyzed. It emerged that the four sensors’ responses to each sample were reproducible, even if each sample was different due to the difference among fecal exhalation composition due to the presence of different gaseous compounds.

Finally, an assessment was also carried out on the response and recovery times of the SmFeO_3_ gas sensors vs. fecal samples. The response time was calculated as the time needed to reach 90% of the steady-state value of the gas sensor response. Recovery time is the range of time that the sensor takes to recover the baseline signal, calculated as the 10% of the response value [40]. These parameters are very important, as is the sensing response value, because all of them are considered in the SCENT prototype algorithm [3]. Therefore, the evaluation of the response and recovery time reproducibility is crucial to validate the reliability of the SmFeO_3_ sensor preparation process. Table 6 and Table 7 show the response (ResT) and recovery times (RecT) of the SmFeO_3_ sensors for the eight fecal samples analyzed.

As can be observed, all the |ResT − <ResT>| values were lower than 8% of ResT, while the |RecT − <RecT>| ones stayed below 7%. These values were considered in the development of the SCENT algorithm used for the clinical trial. The results obtained highlight the high degree of reproducibility of the SmFeO_3_ sensor production process.

This section may be divided by subheadings. It should provide a concise and precise description of the experimental results, their interpretation and the experimental conclusions that can be drawn.

## 4. Conclusions

This work aims to state the reproducibility of responses of chemoresistive gas sensors made of SmFeO_3_ nanostructures, synthesized at the Sensor Laboratory of UNIFE. Reproducibility is fundamental, especially when sensors are employed in mass production of medical devices working with the same analysis algorithm. In particular, SmFeO_3_ is one of the key materials employed in a patented device for CRC preventive screening, already validated through a three-year clinical trial (2016–2019). In this work four identical SmFeO_3_ sensors, obtained from the same synthesis, were tested with three different concentrations of CO, a gas commonly employed for device calibration, in a laboratory setup, and with eight fecal samples in a portable device. Fecal samples came from FOBT-positive subjects, participants in the clinical validation protocol. What emerged from the CO tests was that the value |R − <R>| was lower than 2% of R for 100 ppm of CO and lower than 1% for 50 and 25 ppm of CO. Concerning fecal sample measurements, all |R − <R>| values stayed below 6% of R for all sensors, and below 3% for two fecal samples, demonstrating a high reproducibility degree. The reproducibility of the response and recovery times was also demonstrated for fecal samples. The slight loss in response reproducibility from CO and fecal sample tests could have been due to the huge difference in sample types; the completely different structures of measurement setups—laboratory setup and portable device, respectively; or the employment of different types of airflows, synthetic dry air with the laboratory-test and humidity-stabilized environmental air with the SCENT device. However, despite this negligible difference between the two setups, reproducibility of materials remained high and confirmed the possibility of using this material in medical device sensor arrays.

## Figures and Tables

**Table 1:** Lattice parameters of SmFeO_3_ nanopowder.

a (Å)	5.40077 ± 0.00023
b (Å)	5.59920 ± 0.00024
c (Å)	7.71295 ± 0.00034
Cell Volume (Å^3^)	233.239

**Table 2:** Atomic concentrations (%) of Sm, Fe, O and C in the SmFeO_3_ sample.

Sm (at%)	Fe (at%)	O (at%)	C (at%)
16.67	17.25	56.82	9.26

**Table 3:** Atomic concentrations (%) of Sm, Fe, O and C on the nanoparticle surface of the SmFeO_3_ sample.

Sm (at%)	Fe (at%)	O (at%)	C (at%)
15.5	7.0	33.0	44.5

**Table 4:** Responses R to 25, 50 and 100 ppm of CO; absolute distance from mean value (|R − <R>|); and percentage of R below which the value of |R − <R>| stands for all sensors.

SENSORS	S1	S2	S3	S4
R (100 ppm)	1.39	1.36	1.40	1.38
|R − <R>|	0.00750	0.0225	0.0175	0.00250
2% R	0.0278	0.0272	0.0280	0.0276
R (50 ppm)	1.23	1.24	1.24	1.25
|R − <R>|	0.0100	0	0	0.0100
1% R	0.0123	0.0124	0.0124	0.0125
R (25 ppm)	1.16	1.16	1.17	1.16
|R − <R>|	0.00250	0.00250	0.00750	0.00250
1% R	0.0116	0.0116	0.0117	0.0116

**Table 5:** Responses R to eight FOBT-positive fecal samples; absolute distance from mean value (|R − <R>|); and percentage of R below which the value of |R − <R>| stands for all sensors.

SENSORS	S1	S2	S3	S4
FOBT A	1.24	1.26	1.36	1.26
|R − <R>|	0.0400	0.0200	0.0800	0.0200
6% R	0.0744	0.0756	0.0816	0.0756
FOBT B	2.07	2.04	2.11	2.2
|R − <R>|	0.0350	0.0650	0.00500	0.0950
4% R	0.104	0.102	0.106	0.110
FOBT C	1.51	1.50	1.44	1.60
|R − <R>|	0.00250	0.0125	0.0725	0.0950
6% R	0.0906	0.0900	0.0864	0.0960
FOBT D	1.57	1.55	1.68	1.52
|R − <R>|	0.0100	0.0300	0.100	0.0600
6% R	0.0942	0.0930	0.101	0.0912
FOBT E	2.27	2.24	2.30	2.33
|R − <R>|	0.0150	0.0450	0.0150	0.0450
3% R	0.0681	0.0672	0.0690	0.0699
FOBT F	1.50	1.45	1.55	1.58
|R − <R>|	0.0200	0.0700	0.0300	0.0600
5% R	0.0750	0.0725	0.0775	0.0790
FOBT G	1.73	1.68	1.74	1.76
|R − <R>|	0.00250	0.0475	0.0125	0.0325
3% R	0.0519	0.0504	0.0522	0.0528
FOBT H	1.27	1.24	1.34	1.34
|R − <R>|	0.0275	0.0575	0.0425	0.0425
5% R	0.0635	0.0620	0.0670	0.0670

**Table 6:** Response times (ResT) to eight FOBT-positive fecal samples; absolute distance from mean value (|ResT − <ResT>|); and percentage of ResT below which the value of (|ResT − <ResT>|) stands for all sensors.

	Response Time (s)			
SENSORS	S1	S2	S3	S4
FOBT A	72	75	78	70
|ResT − <ResT>|	1.75	1.25	4.25	3.75
6% ResT	4.32	4.50	4.68	4.20
FOBT B	65	69	67	74
|ResT − <ResT>|	3.75	0.25	1.75	5.25
8% ResT	5.20	5.52	5.36	5.92
FOBT C	103	100	96	92
|ResT − <ResT>|	5.25	2.25	1.75	5.75
7% ResT	7.21	7.00	6.72	6.44
FOBT D	131	135	118	119
|ResT − <ResT>|	5.25	9.25	7.75	6.75
7% ResT	9.17	9.45	8.26	8.33
FOBT E	73	71	77	81
|ResT − <ResT>|	2.50	4.50	1.50	5.50
7% ResT	5.11	4.97	5.39	5.67
FOBT F	93	95	89	86
|ResT − <ResT>|	2.25	4.25	1.75	4.75
6% ResT	5.58	5.70	5.34	5.16
FOBT G	101	96	93	99
|ResT − <ResT>|	3.75	1.25	4.25	1.75
5% ResT	5.05	4.80	4.65	4.95
FOBT H	83	86	81	79
|ResT − <ResT>|	0.75	3.75	1.25	3.25
5% ResT	4.15	4.30	4.05	3.95

**Table 7:** Recovery times (RecT) to eight FOBT-positive fecal samples; absolute distance from mean value (|RecT − <RecT>|); and percentage of RecT below which the value of (|RecT − <RecT>|) stands for all sensors.

	Recovery Time (s)			
SENSORS	S1	S2	S3	S4
FOBT A	227	224	231	211
|RecT − <RecT>|	3.75	0.75	7.75	12.25
6% RecT	13.62	13.44	13.86	12.66
FOBT B	157	153	147	168
|RecT − <RecT>|	0.75	3.25	9.25	11.75
7% RecT	10.99	10.71	10.29	11.76
FOBT C	132	131	121	124
|RecT − <RecT>|	5	4	6	3
5% RecT	6.6	6.55	6.05	6.2
FOBT D	231	220	239	219
|RecT − <RecT>|	3.75	7.25	11.75	8.25
5% RecT	11.55	11	11.95	10.95
FOBT E	182	173	175	167
|RecT − <RecT>|	7.75	1.25	0.75	7.25
5% RecT	9.1	8.65	8.75	8.35
FOBT F	204	209	193	211
|RecT − <RecT>|	0.25	4.75	11.25	6.75
6% RecT	12.24	12.54	11.58	12.66
FOBT G	171	173	166	181
|RecT − <RecT>|	1.75	0.25	6.75	8.25
5% RecT	8.55	8.65	8.3	9.05
FOBT H	214	221	218	208
|RecT − <RecT>|	1.25	5.75	2.75	7.25
4% RecT	8.56	8.84	8.72	8.32
